# Medical Opinion and Movement

**Published:** 1911-06-03

**Authors:** 


### Medical Opinion and Movement.

### Salvarsan.

At a meeting of the Medical Society of Vienna Drs. Ullmann and Handek gave an account of their researches into the elimination of arsenic by the system after injections of dioxydiamidoarseno-benzol. Arsenic may be found at the site of injection three or four months afterwards. The elimination varies according to the muscular force and physical condition of the individual. Intravenous injection causes less local trouble than intramuscular, but in severe cases it is contra-indicated on account of the very lively reaction it may excite. After intravenous injection the arsenic is eliminated fairly rapidly, but traces may still be found at the end of a fortnight. Salvarsan does not act in the same way as arsenic, as has been supposed. It is characterised by a great reducing power, which especially attacks the parasites, to a less extent the cells of the organism, and still less the cellular nuclei. The authors have found arsenic, after salvarsan injections, in the liver, kidneys, stomach, intestines, brain, heart muscles, blood, bones, spleen, and testicles. It is most abundant in the liver and kidneys. But the amount of arsenic found altogether in the organs is relatively small, and it is unknown at present where the surplus of this drug is deposited in the organism. The authors suggest that it is possibly combined with the phenols.

### Hernia in Children.

In a contribution to *Le Journal de Médecine de Paris*, Dr. Savariaud discusses the question of treatment of hernia in young children. He is strongly in favour of operation at quite an early age, even as young as two. He maintains that this is justifiable because with the disinfection of the skin with tincture of iodine, the use of gloves, and the other usual precautions, one can make quite sure of a perfect primary union, and, further, the operation on a young child is so simple that it can be carried out in ten minutes, involving no serious risk of shock to the small patient. According to Dr. Savariaud, the operation can be carried out in this space of time without undue haste and without any attempt at surgical acrobatics. He describes his method of operating for inguinal hernia in young children thus: Having found the hernial orifice with the finger, a skin incision is made over it 4 cm. long. The subcutaneous fat is separated down to the cord. No attempt is made to isolate this by dissection, but by pressing on the scrotum the testicle is made to appear in the wound. This is seized and the cord drawn upon and rapidly isolated with the finger. By stretching the cord between two fingers the sac is quickly recognised by its transparency and whitish colour. This is isolated with the fingers, without the use of any instrument, and then held with fine forceps and dissected out as high up as possible. Two sutures are placed through the pillars of the inguinal ring and three through the skin, and the operation is completed. For children above four years of age the author performs Bassini's operation, but for younger children he considers the method just described sufficient, and it has the advantage of simplicity and rapidity.

### Aviation Sickness.

During the aviation week at Bordeaux last year, Drs. Cruchet and Moulinier questioned the principal aviators in regard to their physical condition, feelings, etc., during flight, and made observations on their blood-pressure before and after flight. The results of their observations were made the subject of a paper read at a recent meeting of the Académie des Sciences. During ascent dyspnœa, tachycardia, noises in the ears, headache, and a pressing need to urinate are experienced. The cold quickly becomes intolerable. These various phenomena are closely similar to those of mountain sickness, but they appear much earlier—with novices at a height of only 500 metres. During descent the same symptoms are experienced with increasing intensity as the aviator approaches the ground, but the dominating troubles are headache and a sensation of burning extending over the whole face, which becomes very congested. There is also a strong inclination to sleep, often compelling the individual momentarily to close the eyes. On reaching the ground the noises in the ears and the desire to sleep are accentuated. There is also frequently vertigo and a sort of heaviness and muscular weakness, with cyanosis of the extremities. The pulse is more rapid and the blood-pressure higher than before the flight. The authors attribute these troubles arising from changes in blood-pressure to the rapidity with which the aviators traverse space. On descending from a height of 1,000, 2,000, or 3,000 metres in four or five minutes, after having attained that height in the course of twenty, thirty, or forty minutes, the organism has not time to adapt its circulatory system to the rapid variations in atmospheric pressure, amounting to 520 mm. of mercury at 3,000 metres, 591 at 2,000 metres, and 760 at the sea-level.

### Gastritis in Infectious Diseases.

Certain gastric troubles, such as anorexia, vomiting, etc., are among the most usual general symptoms of all acute infectious diseases, but they are usually regarded as of a purely functional nature. According, however, to the observations of Dr. Jerusalem, published in the *Deutsche Archiven für Klinische Medizin*, this is by no means the case. He has carefully examined the stomachs of patients who have succumbed to measles, diphtheria, whooping cough, septicæmia, epidemic meningitis, etc., and as a result he finds, excepting whooping cough, all the infectious diseases are accompanied by serious changes in the stomach, usually taking the form of an interstitial gastritis with proliferation of the connective tissue. In the case of whooping cough, the gastric mucous membrane only shows evidence of parenchymatous degeneration, with very slight interstitial changes. Dr. Jerusalem is especially impressed in the course of his research with the uniformity of the anatomo-pathological picture presented by the stomach, contrasting strangely with the great variety of clinical manifestations observed in relation to this organ during life. These observations were carried out at the Pathological Institute of the Augusta Hospital at Berlin.

### Metabolism in the Insane.

R. L. Mackenzie Wallis contributes a paper to the *Journal of Mental Science*, in which he summarises the work that has been done up to date with reference to metabolism in the insane. He finds that: (1) The excretion of creatinine in the insane is generally subnormal, and the creatinine coefficient is correspondingly low; (2) the indigo excreted by the insane appears to be derived from sources other than intestinal putrefaction; (3) the neutral sulphur excretion is low and points to a diminished cellular activity; (4) the results indicate a derangement of cell metabolism in certain forms of insanity, and suggest the administration of glandular extracts known to produce an increase in metabolic changes.

### Hæmorrhagic Intestinal Infarcts.

Hæmorrhagic infarcts of the intestine may be produced by the obliteration either of an artery or of a vein of the mesentery or by an obliteration of both. Two clinical varieties are met with. In the first the infarct is due to arterial obliteration dependent on cardio-vascular lesions and is called "active." In the second the infarction is venous, due to obliteration as the result of thrombo-phlebitis and is called "passive." The former is much the commoner, and usually occurs in the small intestine in the part supplied by superior mesenteric artery. Diagnosis of the condition, though difficult, is possible; the co-existence of an embolus in some portion of the abdomen, cardiac lesions or of some inflammatory process in the intestine or the pelvis may lead to it being suspected. Taravellier, in a "Thèse de Lyon," maintains that the only rational treatment consists in surgical intervention. Laparotomy is always indicated, and though it may prove powerless to help in cases where the lesions are extensive, it is most useful when the latter are circumscribed. The making of an artificial anus is illogical since the dangers of perforation and peritonitis are allowed to persist. The radical operation which has given the best results consists in removal of the infarcted coil with its mesentery, followed by entero-anastomosis. In cases whose general condition is extremely bad it may only be possible to bring the affected coils out of the wound or the two ends of the resected coil. This is a purely palliative proceeding.

### The Dangers of Regimes for the Obese.

Under the title of "The dangers of obesity cures" Dr. Maurice de Fleury presented a communication to the Paris Academy of Medicine in which he states that the first danger of such a cure is the possible lighting up of a latent tuberculosis as the result of too vigorous or of a badly supervised treatment. The cure does not consist merely in getting rid of the fat, but also in the re-establishment of the nerve force, the restoration of the muscles, and the determination of the right kind of food. The second danger is that the cure is often incomplete and insufficient and so gives no permanent result. Patients a few days after abandoning their régime, which can only be of a transitory kind, begin once more to put on fat. Thus, care should be taken when arranging a diet to see that it is one which can become the regular one of the patient after being cured without imposing too much privation upon him.

### Eosinuria in Children.

Eosinuria is by no means uncommon, especially in children who have eaten freely of red sweets that have been coloured with eosin, but it is always alarming both to the patient and his relatives. Dr. A. C. D. Firth has recently recorded two cases in the *Lancet* for May 13, 1911, p. 1276. The first of these was that of a healthy boy aged eleven in whom the mother was greatly alarmed lest acute Bright's disease had supervened owing to the red colour of the urine, which in this case was due to sweets; whilst in the second case the patient was an adult in whom the eosinuria was traced to a red American iced drink and to two red macaroons eaten at the same time. As this patient said, he was "passing a fluid like red ink," and "the condition is certainly very alarming." The diagnosis was suggested at once by the fluorescent appearance of the urine, which looked pink in some lights, greenish in others, and also by the fact that notwithstanding the alarming symptom the general condition of the patient appeared to be perfectly good.

### The Cause of Gastric Ulcer.

After a good deal of careful research work upon animals, Mr. Wilkie has arrived at certain results which seem to bear upon the causation of gastric and duodenal ulceration. Thrombosis of veins of the omentum is, he finds, readily produced by mechanical, thermal, and bacterial agencies. From thrombosed omental veins emboli may frequently separate, probably owing to the periodic vasodilatation and contraction associated with the ingestion of food. Emboli from veins in the omentum may, in certain circumstances, be carried up the gastric veins and become impacted in the venous plexus of the submucous coat of the stomach, and determine a gastric ulcer. So much he has proved. With less confidence he advances certain speculations: that the hæmatemesis sometimes met with in cases of acute appendicitis is due to an acute gastric ulcer resulting from blockage of a gastric vein by a septic embolus from a thrombosed omental vein. That on similar lines the omental vessels may form the connecting link in the association of chronic gastric or duodenal ulcer with an inflammatory process in the lower abdomen. Lastly, that the occurrence of embolism of veins in the pancreas, followed by hæmorrhages into the pancreatic tissue, after the injection of artificial emboli into omental veins, suggests the possibility of portal embolism playing a part in the ætiology of some cases of acute pancreatitis.

### Fibrolysin in Ear Diseases.

The usage and value of fibrolysin for chronic ear diseases are fully discussed by Mr. Macleod Yearsley in the *Journal of Laryngology, Rhinology, and Otology*. He has tried it on twenty patients suffering from a variety of middle-ear complaints, with results which he describes as very disappointing. Three of the patients improved, though even with them he is not absolutely satisfied that the fibrolysin had anything to do with the improvement; the remainder were no better. In only two cases out of seventeen was the symptom of tinnitus relieved. The drug was given by injection in the arm, and although two of the patients complained of pain, not of a lasting character, it was never attended by any objective ill-effects. Whatever may be the action of fibrolysin on cicatricial tissue elsewhere, it has no action, says the author, upon pathological fibrosis in the middle ear occurring as the result of chronic catarrhal inflammation. The whole of the twenty cases are set out in detail: two of them were instances of post-suppurative adhesions, one was otosclerosis with superadded catarrh, and the rest were late stages of chronic middle-ear catarrh or of post-catarrhal adhesions. In every case other treatment had been tried previously. Although Mr. Yearsley has abandoned further trials, he thinks that in a limited number of cases the method may prove of use, but such cases will need to be very carefully selected. He cannot agree, from his own experiences, that fibrolysin merits the eulogies which some observers have lavished upon it.

### Eclampsia and the Wassermann Reaction.

In the *Zeitschrift für Geb. und Gyn.* appears a paper on the examination of the blood of eclamptic patients by Dr. Semon; the paper is summarised in the *British Journal of Obstetrics and Gynæcology*. Among twenty cases of eclampsia, thirteen were severe, six were slight, and one was a case of post-partum amaurosis. His examinations were all made at the height of the symptoms; they revealed three patients whose blood gave a positive Wassermann reaction among the thirteen severe cases, but in all the others the results were negative. One of the positive cases was a very severe one; the patient died after more than a hundred fits, though the urine never showed more than a trace of albumin. Attention to this point was first drawn by Gross and Bunzel in 1909, who found five eclamptic patients giving positive Wassermann reactions at the height of the disease, but not at the beginning or the end. Nadory has also recorded some similar positive cases; but other observers have failed to confirm the results. Exactly what the interpretation of the reaction may be is not easy to say. No one would class eclampsia as a manifestation of syphilis on clinical grounds; but it is, of course, conceivable that syphilitic patients may be more disposed to eclampsia than others.

### Stroganoff's Treatment of Eclampsia.

At Dresden very favourable results have been obtained by what is known as Stroganoff's method of treating puerperal eclampsia. The patient is isolated from everyone except nurse and doctor, and the object is to induce a continual drowsiness or slumberous condition. To begin with, 0.015 gr. of morphia is administered hypodermically. Then under very light chloroform anæsthesia the patient is cleaned up. Half a drachm of chloral hydrate is now given suspended in six ounces of water. The next thing is to await events. If labour is in progress, it is generally ended with forceps at the earliest opportunity; *accouchement forcé* is hardly ever needed or practised. If the patient gets restless, a few drops of chloroform are given; should a fit take place oxygen is applied. Digitalin and camphor are freely used, as also are saline injections. Nothing is allowed by the mouth until the patient regains full consciousness. With this method labour comes on rapidly if it has not already started. In fifteen cases out of fifty fits ceased at once when the treatment began; the maternal mortality is but 8 per cent., and the fœtal only 18 per cent. It is claimed that the plan is far preferable in private practice to any operative measures, as it requires no apparatus or complicated appliances.

### Another Theory of Eclampsia.

The hypothesis of a placental origin of the toxins which may be supposed to be the cause of eclampsia has gained a certain amount of acceptance in this country. Sellheim advances a new explanation of the pathology of this disease. He regards the mammary tissue as the place of origin of the toxins, and he even goes so far as to advocate treatment of eclampsia by amputation of both breasts. Certainly, however, he will have to prove his hypothesis a good deal more satisfactorily before he can expect anyone to imitate this drastic measure, which he has already carried out once. Against the theory of placental origin is the occurrence of eclampsia late in pregnancy, and not when the placenta is first formed or first begins to functionate; also its occasional occurrence after parturition. On the other hand, it always develops about the time the breasts begin to functionate, or when that function is greatly enlarged just after parturition. He suggests the use of "breast gland extract" for eclamptic patients. The milk of eclamptic mothers has been shown to be directly poisonous to the offspring in certain cases, a fact which is decidedly of interest taken in connection with this new hypothesis.

